# Effects of Father and Mother Attachment on Depressive Symptoms in Middle Childhood and Adolescence: The Mediating Role of Emotion Regulation

**DOI:** 10.3390/brainsci11091153

**Published:** 2021-08-30

**Authors:** Alexandra Iwanski, Lucie Lichtenstein, Laura E. Mühling, Peter Zimmermann

**Affiliations:** Human and Social Sciences, Department of Developmental Psychology, Bergische Universität Wuppertal, 42119 Wuppertal, Germany; lichtenstein@uni-wuppertal.de (L.L.); muehling@uni-wuppertal.de (L.E.M.); pzimmermann@uni-wuppertal.de (P.Z.)

**Keywords:** mother attachment, father attachment, depressive symptoms, emotion regulation, mediation

## Abstract

Background: Attachment and emotion regulation play a decisive role in the developmental pathways of adaptation or maladaptation. This study tested concurrent and longitudinal associations between the attachment to mother and father, sadness regulation, and depressive symptoms. Methods: A total of 1110 participants from middle childhood to adolescence completed measures of attachment, emotion regulation, and depressive symptomatology. In total, 307 of them participated in the longitudinal assessment. Results: Results revealed attachment affects emotion regulation strategies and depressive symptoms. Furthermore, we found linear effects of the cumulative number of secure attachment relationships on adaptive and maladaptive deactivating sadness regulation, as well as on depressive symptoms. Longitudinal analysis showed the significant mediating role of sadness regulation in the relationship between attachment and depressive symptoms. Conclusions: Adaptive and maladaptive deactivating sadness regulation explain the longitudinal effects of attachment on depressive symptoms. Insecurely attached children and adolescents use maladaptive and adaptive sadness regulation strategies, but differ in their hierarchy of strategy use.

## 1. Introduction

Attachment theory offers an integrative framework for explaining the role of emotional caregiving experiences in the development of adjustment and psychological wellbeing [1]. The effects of attachment on later adaptation or maladaptation is a central topic of attachment research in childhood and adolescence [2,3,4,5,6,7]. However, as the empirical evidence is mixed, a closer look at the psychological processes connecting attachment and developmental outcomes seems essential [8].

Attachment theory postulates at least three potential mechanisms linking attachment to later wellbeing. First, attachment, as an early-stage salient issue, influences the subsequent development of other competencies [2]. Second, as shown in the field of developmental psychopathology, secure attachment is a main protective factor [9,10,11], whereas insecure attachment is understood as a vulnerability factor, which increases the probability of maladjustment, especially in combination with other risk factors [12,13,14]. Third, early attachment experiences influence the development of internal working models of attachment and the self [1], which control social information processing and emotion regulation and contribute to adjustment or maladjustment [4,15,16]. Emotion regulation patterns developed within child-caregiver relationships are also elicited in other contexts beyond the parent-child relationship, when the individual’s own regulation abilities are overwhelmed [17]. Thus, attachment theory might also be understood as an emotion regulation theory, starting with dyadic and leading towards later individual regulation. The current study is aimed at testing this third potential mechanism, by examining the relationship between attachment and depressive symptomatology, mediated by emotion regulation.

### 1.1. Attachment and Emotion Regulation

Attachment theorists propose that attachment experiences influence later emotion regulation processes, through the development of internal working models of attachment [4,15,16]. From this perspective, attachment patterns represent specific emotion regulation patterns for children, adolescents, and adults: emotional deactivation for avoidant or dismissing attachment, and emotional hyperactivation for ambivalent or preoccupied attachment [15]. However, assessment procedures and the classification criteria differ between infant, adolescent, and adult attachment representation patterns. Moreover, behavioral attachment patterns in infancy do not systematically develop into the complementary attachment representation pattern in adolescence or adulthood [18,19]. In infancy, attachment patterns are classified based on the infant’s behavior when in contact with the caregiver after eliciting distress by separation from the caregiver in the Strange Situation Procedure (SSP) [20]. In adolescence or adulthood, attachment classification in most research is no longer based on attachment behavior, but on the coherence of the reported attachment experiences and their evaluation using the Adult Attachment Interview (AAI) [21,22]. A third attachment research tradition uses self-reports on attachment styles, separating avoidant and anxious attachment or assessing continuous attachment security scores [23,24].

Children that are classified as insecure-avoidant experienced rejection by the attachment figure in their attachment needs. Therefore, they learned a minimizing, deactivating regulation pattern, whereby they do not express distress and attachment needs to the caregiver despite being physiologically distressed [25]. In the SSP, infants with an insecure-avoidant attachment pattern hardly show emotional concern or attachment behavior during separation, and avoid proximity upon reunion, masking their emotional expression. However, this is an ineffective individual emotion regulation, as the suppression of their emotional expression does not regulate their negative emotions sufficiently. Their internal working models of the caregiver include the expectation that the parents are no source of effective regulation and support [15]. Empirical studies support the link between avoidant attachment and emotion regulation in early childhood [26] and beyond. Adolescents classified as dismissing in the AAI showed less adaptive emotion regulation, with a restricted interpretation bias (i.e., low reappraisal), restricted behavior repertoire, and restricted access to own feelings, as well as lower ego-resiliency [16]. Moreover, adolescents with an avoidant attachment style reported less support seeking in adulthood, as shown longitudinally [27].

The theoretical assumption for emotion regulation of children classified as insecure-ambivalent is that they experienced a minimally or inconsistently available attachment figure, and thus developed an emotion regulation pattern of heightened emotional expression [15,28]. In the SSP, insecure-ambivalently attached infants exhibit an intensely distressed reaction to separations, showing both proximity seeking and contact resistance or passivity towards the caregiver after reunion. They are not able to re-establish emotional security and decrease their emotional arousal, despite close proximity to the caregiver. Their emotion regulation pattern is social but ineffective [29]. Some empirical studies support a link between insecure-ambivalent attachment and emotion regulation in early childhood [26,30] and in adolescence. In the AAI, the complementary attachment representation pattern with an assumed similar hyperactivating emotion regulation style is called preoccupied [15]. In the AAI, preoccupied adolescents report contradictory, oscillating evaluations and episodes of the experiences with their caregivers, with passivity of thought or anger. However, they generally value attachment. Adolescents classified as preoccupied in the AAI showed poor adaptive emotion regulation skills, with restricted flexibility of their behavior repertoire and lower ego-resiliency, but no restrictions in access to emotions or appraisal [16]. In the attachment style research tradition, the hyperactivating emotion regulation style is associated with anxious attachment, characterized by concerns that the caregivers will not stay or remain close enough. In studies using attachment style measures, anxious attachment style in adolescence longitudinally predicted more emotion focused regulation strategies in adulthood, such as self-blame, focus on insufficient resources, and anger towards self and others [27].

Securely attached infants in the SSP use the caregiver as a secure base for exploration, show emotional concern and attachment behavior after separation, and are able to gain their emotional stability again by establishing proximity or contact with the caregiver. This has also been shown in middle childhood [31]. Securely attached children show an effective social emotion regulation strategy in an interaction with the caregiver. This effective emotion regulation in interaction with the caregiver seems to transfer into effective self-regulation beyond this relationship. A meta-analysis supports the idea that secure attachment in childhood is associated with effective emotion regulation using cognitive and social support strategies [32]. Adolescents classified as secure in the AAI show coherence in their verbal report about their attachment experiences with both caregivers, freely evaluate positive and negative caregiving experiences, integrate negative attachment experiences and put them into context, and value attachment experiences. Adolescents that are classified as securely attached in the AAI showed more adaptive emotion regulation with flexibility in their evaluations (i.e., reappraisal), behavior repertoire, and access to own feelings, as well as higher ego-resiliency scores. Secure attachment in infancy and secure AAI classification in adolescence were longitudinally and concurrently associated with adolescents’ social support seeking coping and problem solving coping [16,33].

However, these results do not support a clear distinction of deactivating or heightening emotion regulation strategies when comparing individuals classified as insecure-avoidant or dismissing with individuals with an insecure-ambivalent or preoccupied attachment. Nevertheless, the development of emotion regulation from infancy to adolescence leads to an increase in self-regulation.

Therefore, emotion regulation will change in all adolescents independent of their attachment classification in infancy, leading to a general enrichment of their emotion regulation repertoire and knowledge about effective strategy use depending on the specific situation. Thus, adolescents with secure attachment will learn when expressive suppression can be an effective short-term emotion regulation strategy (e.g., presenting in front of a class). Similarly, adolescents with insecure-ambivalent attachment will learn when the use of avoidance or suppression can be effective. However, we assume that there are attachment related differences in the relative use and effectiveness of emotion regulation strategies, leading to attachment related differences in the hierarchy of emotion regulation strategy use.

We therefore assume that dominantly effective social and individual emotion regulation characterizes secure attachment in adolescence. Avoidant attachment is associated with dominant individual and deactivating emotion regulation strategies, which are less effective. Moreover, we assume that ineffective social as well as individual deactivating and hyperactivating emotion regulation characterizes ambivalent attachment in adolescence.

### 1.2. Attachment and Depressive Symptoms

There is ample evidence that attachment security is associated with increased wellbeing, decreased distress in adolescence, and life satisfaction in middle childhood and early adolescence [34,35]. Thus, attachment security is a promotive factor for adjustment and wellbeing.

Insecure attachment is associated with psychological problems, in particular with internalizing symptoms (e.g., depressive symptoms) in children and adolescents [36,37,38,39]. A recent meta-analysis revealed an overall moderate effect size (r = 0.31) for the association between attachment and depression [40]. Longitudinal studies that examine whether attachment insecurity precedes later symptoms and not only correlates with current depression showed that the development of depressive symptoms in later adolescence is predicted by insecure attachment during early adolescence [41], and that an insecure attachment representation predicts higher rates and stable patterns of depressive symptoms across adolescence [5]. Thus, there is a robust, although moderate association between attachment and depression in childhood and adolescence.

### 1.3. Emotion Regulation and Depressive Symptoms

Many studies demonstrate that adolescents with depressive symptoms show an increased use of specific maladaptive emotion regulation strategies (e.g., rumination), as well as a lack of adaptive strategies (e.g., reappraisal) [42,43,44]. Difficulties in emotion regulation, specifically the limited access to emotion regulation strategies, longitudinally predicted an increase in depressive symptoms in early adolescence in boys and girls [45]. A meta-analysis by Schäfer et al. [46] showed that cognitive reappraisal, problem solving, and acceptance were associated with less depressive symptoms, whereas avoidance, suppression, and rumination were associated with increased depressive symptoms in children and adolescents. Thus, adaptive regulation goes along with less symptomatology, whereas maladaptive regulation relates to more symptoms.

However, a closer look at the development of emotion regulation reveals emotion specific differences in the use and effectiveness of emotion regulation strategies in adolescence [47,48]. In the case of depression, especially the prevalence of sadness, is associated with depressive symptoms at that age [49]. Adolescents with internalizing symptomatology showed more deficits in sadness regulation than in fear regulation [50]. Moreover, adolescents’ intensity and lability of sadness, as indicators of deficits in the regulation of sadness, were more strongly associated with depressive symptoms than the intensity and lability of anger and fear [51]. Thus, sadness regulation may play a decisive role in depressive symptomatology.

### 1.4. Attachment, Emotion Regulation, and Depressive Symptoms

Attachment theorists postulate that children adopt specific emotion regulation patterns to deal with distress and negative emotions that are in accordance with their internal working model of attachment [15,52]. Accordingly, individuals use more hyperactivating strategies when faced with distress, in the case of insecure-ambivalent attachment [53]. These regulation strategies serve the goal of maintaining the attention of the attachment figure in the short term, but also lead to intense negative feelings, helplessness, or stress, which in turn contribute to the development of symptoms, including depression, in the long term [54,55]. Rumination and poor mood repair are known as long-term predictors of later depression [56]. Children with avoidant attachment patterns are expected to use deactivating strategies when distressed [4,52]. This may be an effective emotion regulation style in the short term, in the context of rejecting parents, but may contribute to the development of symptoms, including depression or anxiety, in the long-term [57]. This is because negative emotions are suppressed, and mood repair is poorly exercised [58]. Furthermore, attachment hyperactivation may lead to increased susceptibility to internalizing symptoms, whereas attachment deactivation may reduce susceptibility due to differences in threat-related attentional biases [59]. A look at the empirical evidence reveals surprisingly few longitudinal studies examining the relationship between attachment and depressive symptoms. Thus, the mediating or moderating processes and the underlying mechanisms remain unclear.

One mediating factor is emotion regulation [7,39,60]. A review of 19 empirical studies, examining the mediating role of emotion regulation in the relationship between adolescent attachment and depressive symptoms, showed a mixed picture of results [61]. However, the specific assessment of emotion regulation and the specific attachment pattern influenced the mediation. Brenning et al. [39] reported no mediating effect of general dysregulation and suppression for the concurrent association between attachment styles and depressive symptoms. Moreover, attachment styles (anxious and avoidant) were related to depressive symptoms directly and indirectly through sadness regulation strategies as a partial mediator. Brenning and Braet [62] showed an indirect link between an anxious attachment style and concurrent adolescent depressive symptoms through sadness dysregulation. However, the link between attachment avoidance and depressive symptoms was not mediated by sadness regulation. Moreover, Kullik and Petermann [60] reported a mediation by internal dysfunctional regulation, such as self-blaming, of the concurrent association between attachment and depressive symptoms in boys and girls, whereas external dysfunctional regulation, such as venting feelings by shouting, was only a mediator in boys. In addition, repetitive thinking about negative emotions mediated the concurrent association of anxious attachment style and depressive symptoms in middle childhood, but not for avoidant attachment style [63].

Longitudinal data on the association between attachment and depressive symptoms through emotion regulation are rare. Verhees et al. [38] showed that an insecure attachment style was associated with the development of more depressive symptoms, mediated by brooding on negative emotions and a dampening of positive affect in case of anxious attachment style, whereas a reduced focus on positive affect mediated the link between avoidant attachment style and depressive symptoms. Therefore, it is important to investigate emotion regulation, especially sadness regulation, as a possible mediator of the relationship between attachment and depressive symptoms.

### 1.5. Cumulative Effect of Secure Attachments

There is a long debate in attachment research whether the number of secure attachment relationships of a child influence later adjustment [64]. Only few studies investigated whether there are additive or compensatory effects of the number of secure attachment relationships on psychological adaptation. Bretherton [65] suggested an “averaging” effect for children with one secure and one insecure attachment relationship, that may lead to lower wellbeing and adjustment compared to children with two secure relationships. However, having at least one secure attachment may still lead to better adjustment compared to children with two insecure attachment relationships. Verschueren and Marcoen [66] reported some support for this assumption, as children with two secure attachment relationships showed less internalizing symptoms, such as anxious and withdrawn behavior, compared to children with two insecure relationships. Internalizing symptoms in children with only one secure attachment were in between the two other groups, but did not differ significantly. The results may suggest that one secure attachment pattern can compensate for or buffer against the effect of an insecure attachment relationship. The study by Kochanska and Kim [67] also supports the compensation hypothesis, showing that children insecurely attached to both parents reported more behavioral problems than children with at least one secure attachment. However, attachment security with both parents did not result in any additional benefit. Thus, the results did not support a linear cumulative effect of the number of secure attachment relationships on adjustment.

There is a lacuna in this topic, especially in adolescence. We assume, that, the number of secure attachment relationships is a promotive factor for children with regard to symptomatology.

### 1.6. Current Study

The majority of prior studies examining the role of emotion regulation as a potential mediator of the association between attachment and depressive symptoms did not use a longitudinal design, and did not differentiate between attachment to mother and father or a combination in their association with emotion regulation or depression. Studies in middle childhood and adolescence are rare. Moreover, only few studies specifically focused on sadness regulation, a longitudinal predictor of depressive symptoms already at the beginning of adolescence [68]. Thus, we wanted to fill this lacuna, and therefore used a longitudinal approach, including attachment to both parents and sadness regulation. Specifically, we had four aims in our study:

(1) First, we wanted to examine whether adolescents’ attachment security to mother and father is associated with the use of adaptive, maladaptive deactivating, and maladaptive hyperactivating regulation of sadness. We expected more use of adaptive and less use of maladaptive sadness regulation in children and adolescents with secure mother and father attachment, compared to children and adolescents with insecure attachment patterns [15]. We expected that participants classified as insecure-ambivalent would use more maladaptive hyperactivation, and participants classified as insecure-avoidant would use more maladaptive deactivation as sadness regulation, compared to securely attached children and adolescents [69].

(2) Second, we examined the concurrent and longitudinal associations between attachment to mother and father and depressive symptoms. We expected attachment security to both parents to be associated with less depressive symptoms [11].

(3) Third, as extension of the second research question, we were interested in the protective effects of the number of secure attachment relationships on emotion regulation and depressive symptoms. There is no consistent empirical evidence for cumulative or compensation effects of attachment in early childhood [64], and a lack of studies in adolescence. Based on the idea of secure attachment as a promotive and protective factor, we expected a cumulative effect of the number of secure attachments on emotion regulation and depression.

(4) Fourth, we examined the mediating role of sadness regulation in the longitudinal relationship between attachment and depressive symptoms. A core assumption of attachment research is that the effect of attachment security on later adjustment may at least partly be explained by more adaptive and less maladaptive emotion regulation [15,16,32].

## 2. Materials and Methods

### 2.1. Participants

Participants were 1110 German, mainly Caucasian, low risk 5th to 11th graders. Age ranged from 9 to 17 years, with a mean age of 12.94 years (SD = 1.86 years). The sample consisted of an approximately equal number of males and females, and few adolescents who reported being diverse (50.3% females, 49.3% males, 0.5% diverse). Level of education was rather high, with 87.1% of the participants attending a German high school (gymnasium). Other types of schools were secondary modern school (3.5%), comprehensive school (7.7%), and primary school (5th graders, 1.7%). Participants came from schools in North Rhine Westphalia and Berlin, Germany. Participants’ native language was mainly German (64.1%). Despite the different mother tongues of some of the subjects (Turkish 6.8%; Russian 3.8%; Arabian 3.2%; Polish 2.0; Kurdish 1.9%; Albanian 1.1%), all subjects had sufficient knowledge of German to be able to participate in the study. Only participants with written parental consent to participate were involved in the study. From the original sample, 307 subjects (50.8% females, 48.5% males; 0.7% diverse) participated in the longitudinal assessment approximately six months later.

The originally planned longitudinal data assessment with three assessment waves including attachment, emotion regulation, and depressive symptoms (T1; T2: six months later; T3: twelve months later) for the complete sample had to be stopped due to the COVID-19 pandemic during the beginning of wave 2. Thus, drop outs are not due to participants’ retreat. Participants who remained in the study did not differ from drop outs, regarding gender, attachment, and emotion regulation variables. However, drop outs (M = 12.8 years) were slightly younger compared to remaining participants (M = 13.2 years; T(1108) = −3.35, *p* < 0.001, Cohen’s d = −0.23), and showed slightly less depressive symptoms (M = 1.04) compared to remaining participants (M = 1.15; T(1048) = −2.04, *p* = 0.04, Cohen’s d = 0.13). Thus, drop out was not selective for most variables and data for T1 and T2 are rather comparable.

### 2.2. Measures

#### 2.2.1. Attachment-Behavior-Representation Questionnaire (ABR-Q)

Adolescents’ attachment was assessed by use of the Attachment-Behavior-Representation Questionnaire (ABR-Q; [70]), a self-report questionnaire assessing attachment security to mother and father. The ABR-Q measures two main attachment dimensions: attachment behavior strategy (e.g., “I tell my father when I am sad”, mother α = 0.83, father α = 0.89) and attachment representation of parental emotional availability. Attachment representation consists of two subscales: parental perception of adolescents’ distress (e.g., “My mother notices when I am feeling anxious”, mother α = 0.82, father α = 0.88) and parents’ effective support (e.g., “My father helps me when I am sad”, mother α = 0.89, father α = 0.93). The ABR-Q consists of 15 items on attachment processes when experiencing negative emotions, per attachment figure. Adolescents rated each item on a 5-point Likert scale from 1 “never” to 5 ”always”. Higher scores indicate a higher attachment security. Validity has been examined in earlier studies, showing significant associations of ABR-Q with the IPPA ([23]; r = 0.50 and r = 0.80 for mother; r = 0.67 and r = 0.83 for father attachment behavior and representation, *p* < 0.001) and with the ECR ([71]; r = −0.61 and r = −0.67 for avoidance to mother, and r = −0.68 and r = −0.80 for father; and r = −0.13 and r = −0.35 for attachment anxiety to mother, and r = −0.14 and r = −0.32 for father attachment behavior and representation, respectively, *p* < 0.001, except for attachment behavior scales).

For the purpose of the current study, we assigned subjects to three attachment groups, based on the following rationale derived from attachment theory and research in early childhood and adolescence [5,20,29]. Secure attachment in adolescence is characterized by internal working models of parents as emotionally available, supportive, and effective in external regulation. Securely attached adolescents can rely on their parents’ sensitive and effective support when needed, seek their support, and communicate negative affect, but also regulate emotions individually if emotions are not overwhelming. This considers adolescents’ increasing capacity for effective self-regulation, compared to early childhood. Thus, participants with high scores on all attachment subscales (attachment behavior strategy and parental distress perception > 3.0, and effective parental support > 3.5) in the ABR-Q were assigned to the secure group, as well as the subjects with high scores on the subscale of effective parental support (>3.5) and medium scores on the attachment behavior strategy and distress perception subscale (>2.5) (secure group: N = 686 for mother attachment; N = 543 for father attachment). Avoidant attachment in adolescence is characterized by internal working models of parents as emotionally unavailable, rejecting, and not being an effective source of external regulation. We also considered the idealization of the attachment relationship, characterized by positive evaluation of the attachment figures with incoherent examples or lack of memory similar to the dismissing classification in the AAI. Thus, subjects reporting low scores on the attachment behavior strategy scale in the ABR-Q (<2.5) were assigned to the insecure-avoidant group, independently of the parental distress perception and effective support subscale scores (avoidant group: N = 301 for mother attachment; N = 404 for father attachment). Insecure-ambivalent adolescents develop internal working models of their parents as unpredictable, in regards to their perception of the child’s distress, their support, and the effectiveness of their help. Thus, participants with medium scores (range 2.5–3.5) on all three subscales were assigned to the insecure-ambivalent group (ambivalent group: N = 87 for mother attachment; N = 98 for father attachment). Exact algorithms for attachment classification are available from the authors on request. The 36 participants with missing data on mother attachment and the 65 participants with missing data for father attachment were not assigned to one of the attachment groups.

#### 2.2.2. Negative Emotion Regulation Inventory (NERI)

Sadness regulation was assessed by use of the Negative Emotion Regulation Inventory (NERI; [72]), a self-report questionnaire assessing seven regulation strategies in situations of anger, fear, and sadness. The NERI is a reliable and valid measure to assess emotion regulation from late childhood to adulthood [47].

For the current analyses, we used emotion regulation in two sadness situations and aggregated the seven single regulation strategies to three global theoretically derived dimensions for the purpose of the current study: adaptive regulation (α = 0.77), maladaptive deactivation (α = 0.83), and maladaptive hyperactivation (α = 0.81). Adaptive regulation was measured with six items in each sadness situation, with items assessing self-regulation and problem solving. Maladaptive deactivation consisted of the subscales of avoidance (4 items in each situation), passivity (5 items in each situation), expressive suppression (3 items in each situation), and social support seeking (reversed, 4 items in each situation). Maladaptive hyperactivation consisted of the subscales of dysregulation (4 items in each situation) and dysfunctional rumination (5 items in each situation). The respective items were aggregated into mean scores (ranging from 1 to 7, higher scores represent more frequent strategy use) for each of the three dimensions.

Maladaptive deactivation characterizes regulation of sadness by suppressing sad feelings, not communicating sadness, pretending that everything is fine, distracting oneself from the situation or emotion, and passively waiting without seeing any reason to act. Maladaptive hyperactivation characterizes regulation of sadness, by blaming others and oneself, and ruminating about one’s own situation and expectations of others.

#### 2.2.3. Rasch-Based Depression Screening (DESC-II)

We used the Rasch-based depression screening [73] to assess depressive symptoms that were experienced in the past two weeks. The DESC-II consists of ten items. Adolescents completed the DESC-II at both assessments (T1 and T2), and rated each item on a 5-point Likert scale from 0 “never” to 4”always”. The scores were averaged across the ten items to compose a mean score of the DESC-II for T1 and T2 separately (T1: α = 0.86, T2: α = 0.87). Higher scores indicate a higher severity of depressive symptoms. Previous research showed that the DESC-II is a reliable and valid method to screen for depressive symptoms [73,74].

### 2.3. Data Analysis

Data were analyzed with SPSS27. We first report descriptive statistics and zero-order correlations among study variables. Second, ANOVAs for mother attachment and father attachment were performed, to examine the effects of attachment patterns on adolescents’ sadness regulation and depressive symptoms. Third, longitudinal mediation analyses testing the mediating role of sadness regulation for the association between mother and father attachment security (T1) and depressive symptoms (T2) were conducted using PROCESS [75]. Indirect effects were examined using Hayes Model 4 with 5000 bootstrap samples, and biases were corrected at 95% confidence intervals (CI). If the CI did not include zero, it indicates that the effect was significant at *p* = 0.05 [76]. In addition, contrast tests were performed to compare the relative strength between two mediators.

## 3. Results

### 3.1. Preliminary Analyses

First, zero-order correlations between the three father attachment variables: attachment behavior towards father, attachment representation of paternal perception, and attachment representation of paternal effective support were significant positive (all correlations: r > 0.69, *p* < 0.0001). Thus, we computed an overall father attachment security score as a mean of all three attachment dimensions. Similarly, we tested the associations of the mother attachment variables. Again, all mother attachment variables were significantly positively related (all correlations: r > 0.60, *p* < 0.0001). Thus, we computed an overall mother attachment security score as a mean of all three attachment dimensions. Mother and father attachment security scores were also significantly associated (r = 0.70, *p* < 0.0001). However, as mother attachment security (M = 3.60, SE = 0.03) was significantly higher compared to father attachment security (M = 3.27, SE = 0.03), T(1044) = 14.61, *p* < 0.0001, Cohen’s d = 0.45, we used mother and father attachment patterns for the concurrent analyses, and mother and father attachment security for the longitudinal mediation analyses separately.

### 3.2. Descriptive Statistics and Zero-Order Correlations

Table 1 shows means and standard deviations of the study variables, as well as the zero-order correlations. Attachment security was significantly positively related to adaptive regulation of sadness and significantly negatively associated with maladaptive deactivating regulation strategies in sadness situations. Depressive symptoms were significantly negatively associated with attachment and adaptive sadness regulation, and significantly positively related to both maladaptive regulation strategies.

### 3.3. Concurrent Analyses

**Figure 1:**
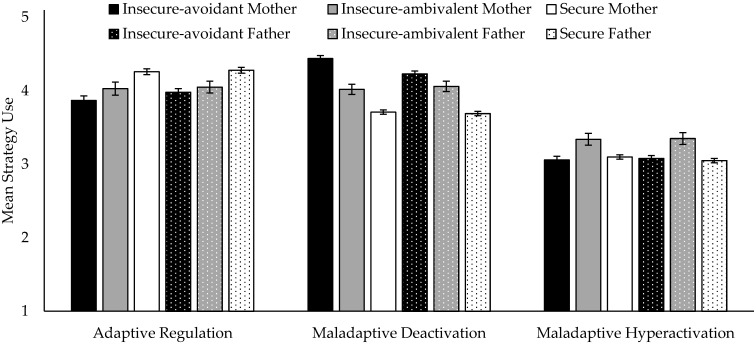
Effects of attachment patterns on sadness regulation. Means and SE for each regulation style.

Examining differences of the attachment groups in emotion regulation, an ANOVA showed a significant effect of mother attachment pattern on adaptive sadness regulation (F(2,1071) = 19.39, *p* < 0.0001, Cohen’s d = 0.42), maladaptive deactivation (F(2,1071) = 107.32, *p* < 0.0001, Cohen’s d = 1.01), and maladaptive hyperactivation (F(2,1071) = 4.20, *p* = 0.015, Cohen’s d = 0.34). Similarly, father attachment pattern also showed a significant effect on adaptive sadness regulation (F(2,1042) = 13.26, *p* < 0.0001, Cohen’s d = 0.33), maladaptive deactivation (F(2,1044) = 63.40, *p* < 0.0001, Cohen’s d = 0.74), and maladaptive hyperactivation (F(2,1044) = 5.45, *p* = 0.004, Cohen’s d = 0.36) (see Figure 1). Duncan post hoc analyses (*p* < 0.05) showed that children and adolescents with secure mother or father attachment reported significantly more use of adaptive sadness regulation strategies compared to both insecure groups. Children and adolescents with insecure-avoidant attachment pattern to mother or father did not differ from insecure-ambivalent participants, regarding their use of adaptive sadness regulation. However, they reported more frequent use of maladaptive deactivating sadness regulation, such as passivity, avoidance, expressive suppression, and no support seeking. Children and adolescents with secure attachment reported significantly less use of maladaptive deactivation compared to both other attachment groups. Children and adolescents with insecure-ambivalent mother or father attachment reported significantly more use of maladaptive hyperactivating regulation strategies, such as dysfunctional rumination and dysregulation, compared to both other attachment groups (see Figure 1).

A pairwise within comparison (all *p* < 0.0001) of sadness regulation strategies for the three attachment groups revealed the following order. Insecure-avoidant mother/father: deactivation > adaptive > hyperactivation; insecure-ambivalent mother/father: deactivation > adaptive > hyperactivation; secure mother/father: adaptive > deactivation > hyperactivation (see Figure 1).

**Figure 2:**
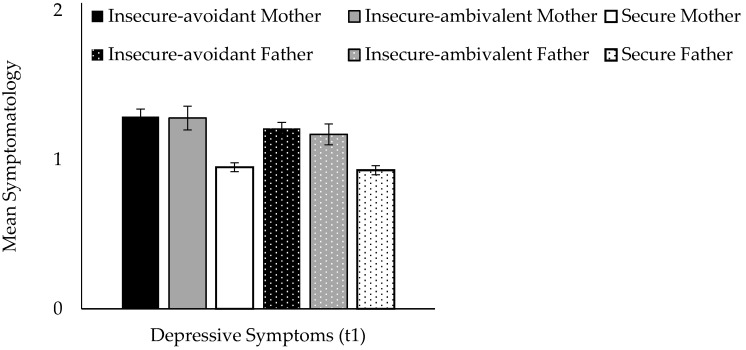
Effects of attachment patterns on depressive symptoms. Means and SE.

Next, we examined the effects of attachment patterns on depressive symptomatology. ANOVAs revealed significant effects of mother attachment pattern (F(2,1045) = 26.00, *p* < 0.0001, Cohen’s d = 0.46), as well as father attachment pattern (F(2,1021) = 19.21, *p* < 0.0001, Cohen’s d = 0.40) on depressive symptoms. Duncan post hoc analyses (*p* < 0.05) showed that securely attached children and adolescents to mother or father reported significantly fewer depressive symptoms compared to both insecure groups, which themselves did not differ significantly (see Figure 2).

**Figure 3:**
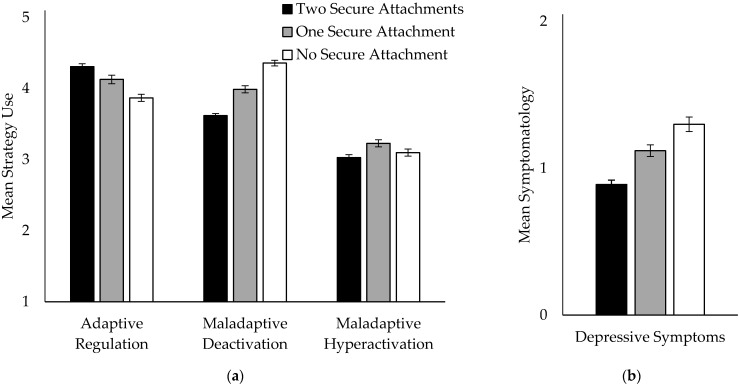
Effects of number of secure attachments on (**a**) sadness regulation (T1) and (**b**) depressive symptoms (T1).Means and SE.

Next, we examined the cumulative effect of the number of secure attachment relationships on emotion regulation of sadness and depressive symptoms. Participants either had no secure attachment (neither mother nor father, N = 332), one secure attachment (mother or father, N = 259), or two secure attachment relationships (N = 485). Subjects who reported to have only one attachment figure (because of death or having no contact) were assigned to the no secure or one secure attachment group, depending on their reported attachment pattern to their attachment figure. An ANOVA revealed a significant effect of the cumulative number of secure attachment relationships on adaptive sadness regulation (F(2,1073) = 22.06, *p* < 0.0001, Cohen’s d = 0.47), maladaptive deactivation (F(2,1073) = 103.02, *p* < 0.0001, Cohen’s d = 1.02), maladaptive hyperactivation (F(2,1073) = 4.66, *p* = 0.010, Cohen’s d = 0.24), and depressive symptoms (F(2,1047) = 30.44, *p* < 0.0001, Cohen’s d = 0.56) (see Figure 3). Duncan post hoc analyses (*p* < 0.05) showed linear differences in the cumulative number of secure attachment relationships. Children and adolescents with two secure attachment relationships to mother and father reported more use of adaptive and less use of maladaptive deactivating sadness regulation strategies, as well as less depressive symptoms, followed by children and adolescents with one secure attachment relationship to mother or father, followed by participants with no secure relationship. In contrast, participants with one secure attachment relationship reported higher scores of maladaptive hyperactivating sadness regulation, compared to children and adolescents with two secure attachment relationships. Children and adolescents with no secure attachment relationship did not differ from both other groups (see Figure 3).

### 3.4. Longitudinal Analyses

**Figure 4:**
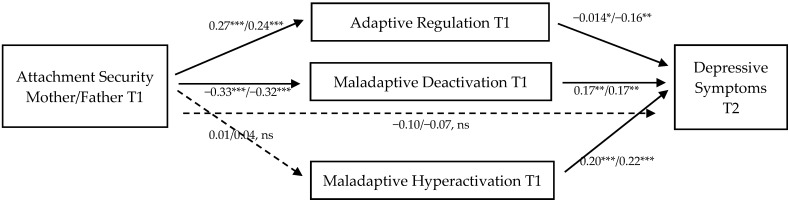
Mediation model of the longitudinal association between attachment security (first coefficient mother attachment security/second coefficient father attachment security) (T1) and later depressive symptoms (T2) mediated by sadness regulation (all path coefficients were standardized). *** *p* < 0.0001, ** *p* < 0.01, * *p* < 0.05.

We examined the mediating role of sadness regulation on the longitudinal association between attachment and depressive symptoms using PROCESS [75]. For mediation analyses, we used the continuous mother and father attachment security scores separately. Results showed rather comparable patterns of direct and indirect effects for mother and father attachment. The mediation models of attachment to each parent are presented in Figure 4 (mother model coefficients/father model coefficients). We included gender as a covariate in the mediation model, as we found significant gender effects on depressive symptoms at T2 (T(302) = −4.96, *p* < 0.0001, Cohen’s d = 0.66) and maladaptive hyperactivating regulation strategies at T1 (T(300) = −2.24, *p* = 0.026, Cohen’s d = 0.57), with female participants reporting higher scores on both scales compared to males.

The mediation analyses revealed significant direct effects of mother and father attachment security on adaptive regulation and the use of maladaptive deactivation, but not on maladaptive hyperactivation. All three sadness regulation dimensions (T1) were significant predictors of depressive symptoms (T2). No significant direct effect of mother or father attachment security (T1) on later depressive symptoms remained in the mediation model (see Figure 4). However, the indirect effects of attachment on later depressive symptoms mediated via adaptive regulation (partially standardized indirect effect mother model: −0.05, 95% CI [−0.090, −0.009]/father model: −0.04, 95% CI [−0.072, −0.008]) and maladaptive deactivation (partially standardized indirect effect mother model: −0.07, 95% CI [−0.124, −0.015]/father model: −0.05, 95% CI [−0.103, −.010]) were significant. Contrast analyses showed that the indirect effects of adaptive regulation (mother model: C1 = −0.05, 95% CI [−0.101, −0.001]/father model: C1 = −0.05, 95% CI [−0.090, −0.006]) and maladaptive deactivation (mother model: C3 = 0.07, 95% CI [0.008, 0.134]/father model: C3 = −0.06, 95% CI [0.009, 0.118]) were significantly stronger than the effect of maladaptive hyperactivation on depressive symptoms. Thus, the effect of attachment security to mother and father on later depressive symptoms is mediated by adaptive sadness regulation and maladaptive deactivation, but not by maladaptive hyperactivation.

## 4. Discussion

The main goals of this study were to examine whether attachment is associated with emotion regulation of sadness and depression in middle childhood and adolescence, and whether emotion regulation would explain the longitudinal association between attachment and depressive symptoms.

Bowlby [1] proposed that secure attachment is characterized by internal working models of one’s own caregivers as emotionally available, effectively supportive, and by seeking proximity when experiencing distress that exceeds one’s own regulatory capacities. Consequently, secure attachment will be associated with effective emotion regulation, and insecure attachment with more maladaptive and ineffective emotion regulation [15,16]. However, when differentiating insecure attachment patterns, attachment theorists propose completely different emotion regulation patterns. While insecure-avoidant or dismissing attachment is expected to be associated with more deactivating emotion regulation, insecure-ambivalent or preoccupied attachment is expected to be associated with more hyperactivating regulation [15,52]. The results of the association between attachment and emotion regulation depend on whether attachment is assessed as one continuous security dimension or as attachment patterns (secure, avoidant, and ambivalent).

We found that children and adolescents’ attachment security to parents was associated with the use of more adaptive and less maladaptive deactivating sadness regulation, but not with less maladaptive hyperactivating sadness regulation. However, differentiating between attachment groups, securely attached children and adolescents used more adaptive and less maladaptive deactivating sadness regulation than both insecure attachment groups. Interestingly, and in line with the ideas of many attachment theorists, the insecure-ambivalent attachment group used more hyperactivating sadness regulation than the other attachment groups. Thus, they reported more rumination, and blaming of themselves and others when feeling sad. However, insecure-ambivalently attached children and adolescents also used deactivating emotion regulation strategies for sadness, just as participants with insecure-avoidant classification. This is in line with earlier studies showing that both dismissing and preoccupied AAI scores are similarly associated with avoidant coping or ego-resiliency [16,77,78]. Furthermore, studies using attachment style measures show comparable results [79]. Therefore, these results might broaden our perspective on the association between attachment and emotion regulation. Insecurely attached children and adolescents use both adaptive and maladaptive sadness regulation strategies and not only either deactivating or hyperactivating strategies. However, attachment groups may well differ in their respective hierarchy of emotion regulation strategy use. In our study, securely attached children and adolescents mainly used adaptive sadness regulation, compared to their use of the two maladaptive regulation styles. Insecure-avoidant attached children and adolescents most often used maladaptive deactivation, followed by adaptive regulation, and least used maladaptive hyperactivation. In contrast, a comparably frequent use of deactivating and adaptive regulation and a subdominant use of maladaptive hyperactivating sadness regulation characterized the hierarchy of insecure-ambivalently attached individuals. This supports the perspective that insecure attachment is no disorder, but may increase children’s’ vulnerability to the development of later maladjustment [3,9]. In a similar vein, children and adolescents with insecure attachment patterns are not only using maladaptive emotion regulation strategies but also to a lesser extent using adaptive regulation strategies. Moreover, and extending other studies, we found similar associations with emotion regulation of sadness for attachment classifications to mother and father, showing that at least for that age group attachment to both parents is relevant for emotional competence.

The second main finding of the study is that attachment security as a dimensional score was concurrently and longitudinally associated with depressive symptoms comparable to earlier studies [5,39,80]. Moreover, children and adolescents classified as insecure-avoidant attached or as insecure-ambivalent attached reported more depressive symptoms, compared to securely attached children and adolescents. This is in line with earlier studies on mother attachment [81,82]. However, in this study, both attachment to mother and father show a comparable effect. Although the results are in line with earlier studies, this study shows a somehow lower effect size compared to the mean effect size reported in meta-analyses [37].

Earlier research emphasized that only longitudinal associations between attachment and depression allow to appropriately exploring the mediating mechanisms via emotion regulation [39]. Our study showed that adaptive sadness regulation and maladaptive deactivating regulation of sadness were both longitudinal mediators of the association between attachment security to parents and later depressive symptoms. The results are consistent with the idea that secure attachment has a promotive effect on adolescents’ ability to regulate negative emotions effectively, which is a competence for later adjustment [4,15,16,28]. Showing attachment behaviors towards caregivers when emotionally challenged in adolescence, and having an internal working model of both caregivers as emotionally available and supportive does not promote dependency in adolescents, but increases their autonomous and effective emotion regulation, which in turn leads to fewer depressive symptoms. Thus, secure attachment may be a developmental precursor of emotion regulation strategies that are clinically relevant for adjustment and low depressive symptomatology in childhood and adolescence [46]. This study at least shows that attachment and sadness regulation are concurrently associated. In contrast, the vulnerability for maladaptation associated with insecure attachment in middle childhood and adolescence may be a result of the development of a hierarchy of emotion regulation strategies, where maladaptive strategies such as avoidance, passivity, suppression, and social withdrawal are used more frequently than adaptive strategies. Frequent use of maladaptive emotion regulation strategies has shown to be associated with internalizing symptoms in adolescence and adulthood [46,83]. Nevertheless, we want to emphasize that children and adolescents with insecure attachment also use adaptive emotion regulation strategies.

The current study did not show a mediation from the dimensional attachment security score to depression via hyperactivating emotion regulation. This contradicts existing theoretical assumptions, proposing that hyperactivation in the domain of attachment is transferred to hyperactivation of emotion regulation in other domains, leading to depression. Although this hypothesis has face validity, the empirical evidence is mixed and suggests that the ambivalent or preoccupied attachment pattern is also associated with the use of deactivating emotion regulation [77,78,84]. We assume that the ambivalent attachment pattern changes in form from infancy to middle childhood and adolescence, similar to that of infant disorganization, which develops into later controlling behavior [85]. Insecure-ambivalent attachment in middle childhood and adolescence seems no longer solely characterized by heightened arousal, hyperactivation, and a poorly regulated need for proximity, but by a mixture of proximity seeking and individual emotion regulation attempts, such as avoidance, suppression, or passivity that still—and perhaps comparable to infancy—do not effectively regulate emotions.

The role of passivity as an emotion regulation strategy of insecure-ambivalently attached adolescents and older children needs further exploration in future studies. Passivity, as assessed in the NERI, is a deliberate strategy, whereas passivity in the SSP or the AAI may represent helplessness or low meta-monitoring as signs of a lack of emotion regulation, but not as a deliberate strategy to deal with the emotions.

Finally, we found evidence for the cumulative attachment relationship hypothesis [64,65]. The results suggest a linear effect for middle childhood and adolescence. The more secure attachment relationships, the fewer depressive symptoms. It may well be that this is a developmental period, where attachment to father might have more effect compared to earlier age periods, leading to this cumulative effect. In longitudinal studies, we found infant attachment to mother but not to father was associated with adjustment in childhood but not adolescence [86]. In contrast, in adolescence, infant father attachment but not infant mother attachment predicted adolescents’ coping or social interaction with friends [17,77]. However, future studies need to replicate such cumulative attachment effects.

Although the current study yielded some important and unique findings, it also has limitations. First, all measures used in the study were self-report measures, which may be affected by social desirability. In addition, common method variance can lead to an overestimation of associations. Moreover, the assessment of attachment security and attachment pattern in older children and adolescents, using the ABR-Q, is introduced in the current study for the first time, as well as the two maladaptive NERI emotion regulation styles of maladaptive deactivation and maladaptive hyperactivation. Thus, further replication and validation is needed. Results of the ABR-Q showed a high association of mother and father dimensional attachment security scores, and this may raise the question whether they represent distinct or overlapping concepts. However, other studies on older children and adolescents also show high associations of attachment security to mother and father in questionnaires (e.g., [11,12]) or in child attachment interviews [87,88]. Moreover, attachment security scores to mother were higher compared to the attachment security scores to father. The classification approach showed that 25% of the sample only showed one secure relationship to either mother or father. We conclude that dimensional attachment security to mother and father are not distinct but related constructs. However, differences in attachment patterns to mother and father are not a rare exception at this age.

Additionally, as shown in the meta-analysis by Madigan et al. [37], associations between attachment and symptomatology vary depending on construct measurement. Questionnaire measures of attachment revealed stronger associations with internalizing symptoms compared to representational measures (such as the AAI or attachment narratives) or even behavioral measures (i.e., SSP or the Attachment Q-Sort). Future research may add observational or representational measures of attachment and symptom assessments by other informants (e.g., parents, teachers, or clinicians).

The participants in this study were nonclinical, mainly Caucasian community sample children and adolescents, which limits generalization of our findings regarding symptom severity. Future research may use a clinical sample to increase variance in depressive symptoms. However, even 52% of our nonclinical sample had scores higher than the clinical cut-off of the DESC-II at T2, which does not yet imply a diagnosis, but an increased probability of severe clinical symptoms.

A great advantage of the current study is the longitudinal design, which allows the examination of developmental mechanisms. However, due to the COVID-19 pandemic, we had to stop the originally planned three-wave assessment of attachment, emotion regulation, and depressive symptomatology. Thus, the presented mediation only includes the parallel assessment of attachment and emotion regulation at T1 as predictors of later depressive symptoms at T2. This is a clear limitation of this report, as we cannot completely examine the assumed developmental mechanism of attachment security as a precursor of later emotion regulation influencing later depressive symptoms. Thus, future studies should use more assessment waves, a longer period for the prediction of depressive symptoms, and examine potential stabilizing processes of emotion regulation [89]. Cross-lagged panel analyses may help to detect the direction of associations between attachment, emotion regulation, and depressive symptoms during adolescence, as the cross-lagged effects may not be evident during all phases of adolescence [80].

Developmental psychopathology postulates the promotive and protective effect of attachment security for later development [11]. Our study supports the promotive effect of attachment security, due to our non-risk sample. However, this does not allow final conclusions about the protective effect of attachment security. Future studies should address this developmental psychopathological idea using a risk sample.

## 5. Conclusions

Adaptive and maladaptive emotion regulation are longitudinal mediators of the effect of attachment security on later depressive symptoms in middle childhood and adolescence. Moreover, children and adolescents with insecure attachment patterns do not show only deactivating or hyperactivating emotion regulation. On the contrary, they all use several emotion regulation strategies and adaptive regulation. However, the hierarchy of emotion regulation use differs between attachment groups, explaining discrepancies in developmental outcomes.

## Figures and Tables

**Table 1:** Means, standard deviations, and correlations of all variables, age, and gender at time point 1 and follow-up.

		1	2	3	4	5	6	7	8	9
Time Point 1										
	**Attachment:**									
	1. Attachment Security to Mother	-								
	2. Attachment Security to Father	0.70 ***	-							
	**Emotion Regulation of Sadness:**									
	3. Adaptive Regulation	0.20 ***	0.18 ***	-						
	4. Maladaptive Deactivation	−0.40 ***	−0.31 ***	0.02	-					
	5. Maladaptive Hyperactivation	−0.00	−0.02	−0.08 **	−0.03	-				
	**Symptomatology:**									
	6. Depressive Symptoms	−0.26 ***	−0.22 ***	−0.20 ***	0.21 ***	0.34 ***	-			
Follow-Up (T2)										
	**Symptomatology:**									
	7. Depressive Symptoms	−0.15 **	−0.14 **	−0.18 **	0.16 **	0.23 ***	0.56 ***	-		
Demographic Variables										
	8. Age (T1)	−0.26 ***	−0.27 ***	0.10 **	0.21 ***	0.02	0.13 ***	-	-	
	9. Age (T2)	-	-	-	-	-	-	0.12 *	-	-
	10. Gender	0.15 ***	0.06	−0.10 **	−0.08 **	0.18 ***	0.20 ***	0.29 ***	−0.05	−0.12 *
*M*		3.59	3.27	4.13	3.93	3.10	1.07	1.29	12.94	13.77
*SD*		0.81	1.00	0.95	0.79	0.84	0.75	0.79	1.86	1.70

Note. Gender is dummy coded (male = 1, female = 2); age is cross-sectional (at time point 1 for time point 1 variables and at follow-up for follow-up variables). *** *p* < 0.001, ** *p* < 0.01, * *p* < 0.05.
